# Treating Glioblastoma Multiforme (GBM) with super hyperfractionated radiation therapy: Implication of temporal dose fractionation optimization including cancer stem cell dynamics

**DOI:** 10.1371/journal.pone.0245676

**Published:** 2021-02-01

**Authors:** Victoria Y. Yu, Dan Nguyen, Daniel O’Connor, Dan Ruan, Tania Kaprealian, Robert Chin, Ke Sheng

**Affiliations:** Department of Radiation Oncology, David Geffen School of Medicine, University of California Los Angeles, Los Angeles, California, United States of America; Sechenov First Medical University, RUSSIAN FEDERATION

## Abstract

**Purpose:**

A previously developed ordinary differential equation (ODE) that models the dynamic interaction and distinct radiosensitivity between cancer stem cells (CSC) and differentiated cancer cells (DCC) was used to explain the definitive treatment failure in Glioblastoma Multiforme (GBM) for conventionally and hypo-fractionated treatments. In this study, optimization of temporal dose modulation based on the ODE equation is performed to explore the feasibility of improving GBM treatment outcome.

**Methods:**

A non-convex optimization problem with the objective of minimizing the total cancer cell number while maintaining the normal tissue biological effective dose (BED_normal_) at 100 Gy, equivalent to the conventional 2 Gy × 30 dosing scheme was formulated. With specified total number of dose fractions and treatment duration, the optimization was performed using a paired simulated annealing algorithm with fractional doses delivered to the CSC and DCC compartments and time intervals between fractions as variables. The recurrence time, defined as the time point at which the total tumor cell number regrows to 2.8×10^9^ cells, was used to evaluate optimization outcome. Optimization was performed for conventional treatment time frames equivalent to currently and historically utilized fractionation schemes, in which limited improvement in recurrence time delay was observed. The efficacy of a super hyperfractionated approach with a prolonged treatment duration of one year was therefore tested, with both fixed regular and optimized variable time intervals between dose fractions corresponding to total number of fractions equivalent to weekly, bi-weekly, and monthly deliveries (n = 53, 27, 13). Optimization corresponding to BED_normal_ of 150 Gy was also obtained to evaluate the possibility in further recurrence delay with dose escalation.

**Results:**

For the super hyperfractionated schedules with dose fraction number equivalent to weekly, bi-weekly, and monthly deliveries, the recurrence time points were found to be 430.5, 423.9, and 413.3 days, respectively, significantly delayed compared with the recurrence time of 250.3 days from conventional fractionation. Results show that optimal outcome was achieved by first delivering infrequent fractions followed by dense once per day fractions in the middle and end of the treatment course, with sparse and low dose treatments in the between. The dose to the CSC compartment was held relatively constant throughout while larger dose fractions to the DCC compartment were observed in the beginning and final fractions that preceded large time intervals. Dose escalation to BED_normal_ of 150 Gy was shown capable of further delaying recurrence time to 452 days.

**Conclusion:**

The development and utilization of a temporal dose fractionation optimization framework in the context of CSC dynamics have demonstrated that substantial delay in GBM local tumor recurrence could be achieved with a super hyperfractionated treatment approach. Preclinical and clinical studies are needed to validate the efficacy of this novel treatment delivery method.

## I. Introduction

Glioblastoma multiforme (GBM) is a devastating primary brain cancer with abysmal survival rates. Approximately 12,000 people are newly diagnosed with GBM each year in the United States alone, accounting for more than 51% of all brain gliomas, making it the most common type of primary brain tumor [1–3]. Even with surgical resections followed by radiotherapy and chemotherapy, predominantly local recurrence occurs and the overall median survival is still only 14 months [2, 4, 5]. Aside from the conventionally utilized fractionation schemes of 1.8 Gy × 33 and 2 Gy × 30, numerous alterations in dose fractionation and escalation schemes were attempted in hopes to improve treatment outcome and reduce treatment duration. Accelerated hyper-fractionated twice a day (b.i.d) delivery of 1 to 1.5 Gy fractions 2 or 3 times a day [6–10], accelerated dosing of multiple 2 Gy fractions a day [11–13], hypofractionated 3 to 6 Gy [14–17] dosing schemes and aggressive dose escalation to 110 Gy using combined regularly fractionated external beam therapy and low dose rate (LDR) brachytherapy [18, 19] were implemented with no significant benefit in overall survival or durable local control [20]. Greater incidence of brain necrosis was found in the hypo-fractionated and aggressive dose escalation approaches. Although the toxicity was not increased in the hyper-fractionated and accelerated -radiotherapy methods, the benefit of reducing treatment time alone did not gain sufficient support for a paradigm shift.

In the meantime, fractionation schedule optimization (FSO), the method of systematically deriving the most effective fractionation schedule that maximizes biological effective dose (BED) to the tumor while maintaining acceptable toxicity to surrounding normal tissue, has been actively investigated. With fixed time intervals of once or twice per weekday, Wein et al. demonstrated that up to two time increase in tumor control probability could be achieved by utilizing larger fractions before overnight and weekend breaks [21]. A dynamic programming framework in the presence of tumor repopulation was established to determine the optimal dose delivery schedule, which suggested a gradual increase in fraction size throughout the treatment course can improve tumor control by up to 50% [22]. Lindblom et al. studied the effectiveness of varying fractionation for non-small cell lung cancer based on the concept of heterogeneous spatial and temporal oxygenation, differing effects of accelerated repopulation, and intra-fraction repair. The study revealed that schedules with a baseline fractional dose of 2 Gy accompanied by an escalation to 3 or 4 Gy per fraction could improve the tumor control probability by up to 3 fold [23]. Kim et al. showed that the tumor equivalent uniform dose (EUD) may be increased by 17% using spatiotemporal optimization [24]. Therefore, it is interesting to test the efficacy of FSO on GBM tumors. To test FSO, the unique radiobiological properties of GBM need to be considered.

Despite the extreme radioresistance demonstrated in patients, GBM cell lines do not appear to be particularly radioresistant *in vitro*. Instead, an extremely wide range of intrinsic radiosensitivities largely overlapping with the *in vitro* survival results of tumors curative with radiation was observed [25–27]. This discrepancy suggests that the aggressive tumor behavior of GBM cannot be adequately reflected with the simple classical radiobiological models that assume a tumor cell population with uniform radiosensitivity and radiobiological characteristics. A recently proposed ordinary differential equation (ODE) model that took into consideration the dynamic interaction and distinct radiosensitivity between cancer stem cells (CSC) and its non-stem counterpart, differentiated cancer cells (DCC), was shown capable of describing the definitive treatment failure of GBM based on human GBM cell parameters [28–30]. A mathematical model of PDGF-driven glioma with consideration of heterogeneous tumor subpopulations was utilized in an iterative combined theoretical and experimental strategy and identified two hyper-fractionated schedules within a five day treatment period that led to superior survival in mice [31]. These studies clearly show the potential of substantially delaying GBM recurrence without increasing normal tissue toxicity. Thus, the goal of this study is to develop a temporal dose fractionation optimization framework with consideration of CSC dynamics in an effort to discover dosing schemes with the potential to significantly delay GBM recurrence. A novel super hyperfractionated approach which was discovered through the creation of an optimization formulation will also be introduced.

## II. Methods

The methodology of this study will be introduced in four major components. First, the ODE model utilized to describe the dynamic interaction between the CSC and DCC compartments. Second, the workflow of simulating radiation therapy along with ODE tumor growth. Third, the temporal dose fractionation optimization problem formulated specifically for the proposed tumor growth and radiation killing model followed by the utilized algorithm will be demonstrated. Lastly, the optimization scenarios and conditions applied, including conventional time frames and a prolonged super hyperfractionated approach will be described.

### II.1. ODE model

The ODE model used to simulate the dynamic interaction and growth of CSC and DCC is shown in the Eq 1 below:

$$\begin{array}{lll} & \text{Self}-\text{renewal} & \\ \dot{U}(t)= & \left(2P-1)m_{U}k(W(t))U(t\right) & \\ \dot{V}(t)= & 2(1-P)m_{U}k(W(t))U(t) & +m_{V}k(W(t))V(t)-a_{V}V(t)\\ & \text{Differentiation}\;\text{from}\;\text{CSC} & \text{DCC}\;\text{growth}\;\text{DCC}\;\text{natural}\;\text{cell}\;\text{death}\\ W(t)= & U(t)+V(t) & \\ k(W) & \text{max}[1-W^{4},0] & \end{array}$$ (1)

where U(t), V(t), and *W*(*t*) represent the volume fractions of CSCs, DCCs, and total tumor with respect to a specified volume of interest in which the tumor can grow. The model assumes no asymmetric divisions, where one CSC gives rise to either two CSCs or two DCCs, with probabilities of *P* and 1-*P*, respectively. The growth rates of CSC and DCC are *m*_*U*_ and *m*_*V*_, and *a*_*v*_ is the natural cell death rate of DCCs. Following previous publications [28, 29], all three parameters were set to ln(2)/T_pot_ day^-1^, where T_pot_ represents the tumor potential doubling time of malignant brain tumors [32]. α and β parameters of the CSC and DCC compartments were obtained by performing dual compartment model data fit on clonogenic survival data of a GBM U373MG cell line [33], as detailed in a prior publication [28]. *k(W)* is a monotonically decreasing volume constraint function that keeps the total tumor volume fraction (*W*) within the range of 0 and 1 while simulating the slowdown in growth rate as new born cells compete for resources within the available growth volume [30]. All simulations in this study were set to have the specified volume of interest to be 10^11^ cells. The simulation was initialized with a tumor volume of 1.8 × 10^9^ cells, corresponding to a postoperative mean T1 post-gadolinium enhancement volume of 1.8 ml from 721 patients [34]. As patients typically receive radiation thirty days after surgery (range, 3–6 weeks) [35], the ODE was utilized to simulate 30 days of tumor growth with no treatment intervention from the specified initial conditions prior to starting radiation therapy. The ODE simulation parameters are summarized in Table 1.

**Table 1:** ODE simulation and optimization parameters.

**ODE parameters**				**Radiation therapy parameters**					
***N***_***Tumor***_	***m***_***U***_	***m***_***V***_	***a***_***V***_	***F***	***α***_***CSC***_	***β***_***CSC***_	***α***_***DCC***_	***β***_***DCC***_	***c***
1.80E-02	0.1777	0.1777	0.1777	0.016	0.01	1.77E-07	0.125	0.028	5.196E-03

**Universal optimization parameters**										
***μ***	**λ**	***R***	***s***_***D***_	***s***_***T***_	***T***_***prob***_	***T***_***step***_	***L***_***S***_	***D***_***max***_	***D***_***min***_	***r***
10	10000	2.8E-02	40	15	1	2	1	15	1*	2

*exception in b.i.d schedules.

### II.2. Modeling radiation therapy

The distinct radiosensitivity of the CSC and DCC compartments specific to GBM were determined by performing curve fitting on clonogenic cell survival data with a dual-compartment linear quadratic (DLQ) model [28], as shown in Eq 2.

$$SF(D)=F\cdot \text{exp}\{-\alpha_{CSC}D-\beta_{CSC}D^{2}+(1-F)\cdot \text{exp}\{-\alpha_{DCC}D-\beta_{DCC}D^{2}\},$$ (2)

with *F* as the fraction of CSC out of all tumor cells, and *α*_*CSC*_, *β*_*CSC*_, *α*_*DCC*_, and *β*_*DCC*_ describing the radiobiological properties corresponding to the CSC and DCC compartments.

**Fig 1:**
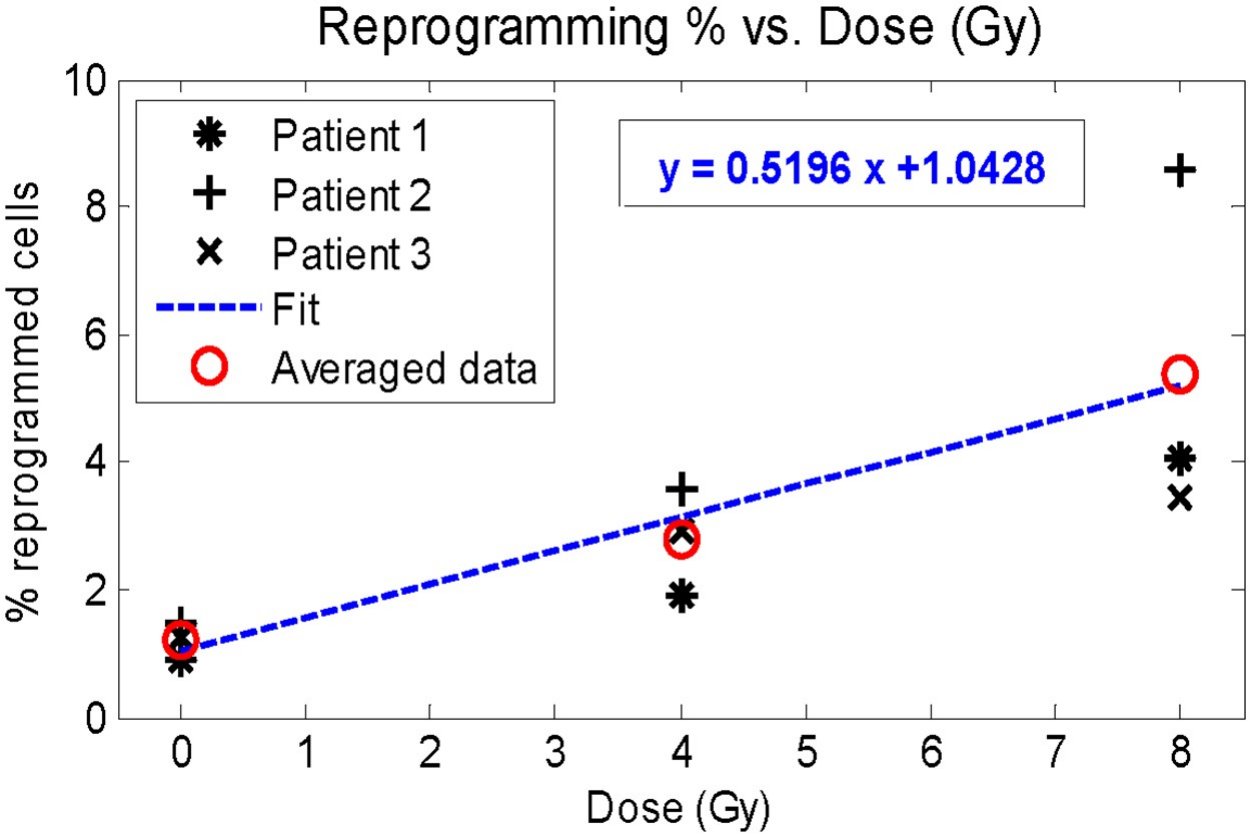
Percentage of radiation-induced DCC reprogramming to CSC with respect to received dose. Determination of reprogramming coefficient *c* with linear regression.

**Fig 2:**
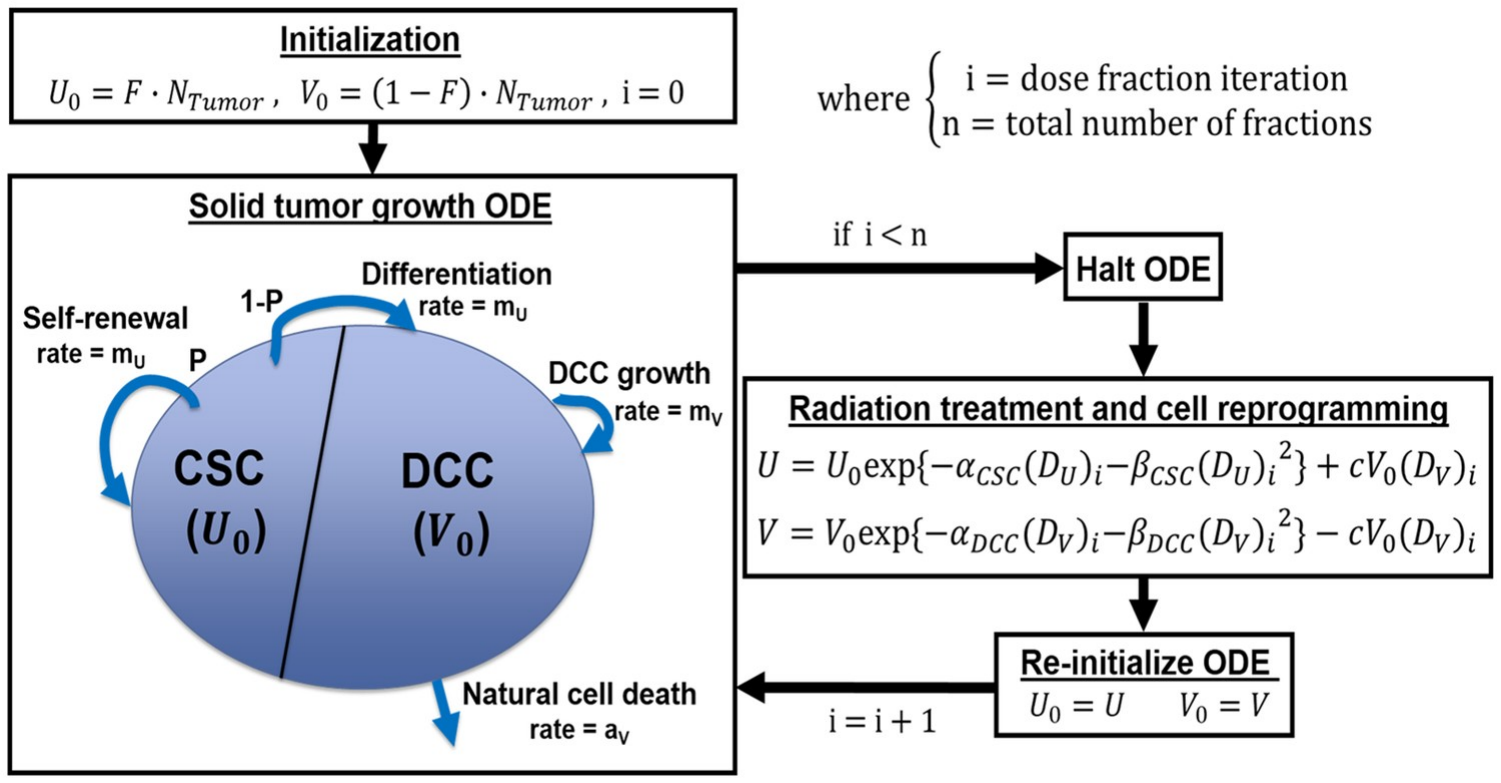
Tumor growth ODE and radiation therapy simulation schematic.

Furthermore, there is recent evidence suggesting that a fraction of DCC reprograms back into CSC after radiation exposure and the reprogramming rate is proportional to the dose received [36, 37]. A new reprogramming term linear to dose was therefore incorporated into the model. Linear quadratic radiation therapy cell killing and reprogramming to both compartments are applied as follows in Eq 3:

$$\begin{array}{ll} U(t)= & U_{0}\text{exp}\{-\alpha_{CSC}{\left(D_{U}\right)}_{i}-\beta_{CSC}{\left(D_{U}\right)}_{i}{}^{2}\}+cV_{0}{\left(D_{V}\right)}_{i}\\ V(t)= & V_{0}\text{exp}\{-\alpha_{DCC}{\left(D_{V}\right)}_{i}-\beta_{DCC}{\left(D_{V}\right)}_{i}{}^{2}\}-cV_{0}{\left(D_{V}\right)}_{i} \end{array},$$ (3)

where *U*_*0*_ and *V*_*0*_ are the compartmental cell fractions after halting the ODE at dosing time points, (***D***_***U***_)_***i***_ and (***D***_***V***_)_***i***_ are the radiation delivered to CSC and DCC on the ith fraction, and *c* is the reprogramming coefficient. The model assumes that the subvolumes with enriched CSC could be localized and targeted by a simultaneous integrated boost dose. If this is not achievable, then our solution would be limited to the special case that ***D***_***U***_ = ***D***_***v***_. After applying the radiation therapy term shown in Eq 3, the ODE resumes 0.01 days (14.4 minutes) after the treatment time point. The dose dependent reprogramming coefficient *c* was determined based on linear regression on percentage of radiation-induced CSC from purified non-stem human breast cancer specimens with respect to dose [36]. The data and corresponding linear fit is demonstrated in Fig 1, where both patient specimen specific and averaged patient data are shown. The fitting was performed using the average of all three patient derived data sets, indicated with red circles. The resultant slope from the linear regression was utilized as the reprogramming coefficient in simulations. A full schematic of the tumor growth and radiotherapy simulation is shown Fig 2.

### II.3. Optimization formulation and algorithm

The formulated optimization problem is shown in Eq 4 below:

$$\begin{array}{c} \underset{D_{U},D_{V},T}{\text{argmin}}\;W(t_{eval}|D_{U},D_{V},T)+\mu \frac{\sum_{t=0}^{L}(L-t)W(t|D_{U},D_{V},T)}{\sum_{t=0}^{L}(L-t)}+\lambda \overset{L}{\underset{t=0}{\sum }}\text{max}(W(t|D_{U},D_{V},T)-R,0)\\ \text{subject}\;\text{to}\;\sum_{i=1}^{n}{\left(D_{U}\right)}_{i}+\frac{{\left(D_{U}\right)}_{i}{}^{2}}{\alpha /\beta }\leq {\text{BED}}_{U},\;\sum_{i=1}^{n}{\left(D_{V}\right)}_{i}+\frac{{\left(D_{V}\right)}_{i}{}^{2}}{\alpha /\beta }\leq {\text{BED}}_{U},\\ D_{min}\leq D_{U},D_{V}\leq D_{max},\;\sum_{i=1}^{n-1}T_{i}=L,L_{s}\leq \text{T}\leq L,\;\frac{1}{r}\leq \frac{{\left(D_{U}\right)}_{i}}{{\left(D_{V}\right)}_{i}}\leq r\;for\;i=1..n \end{array}$$ (4)

**Fig 3:**
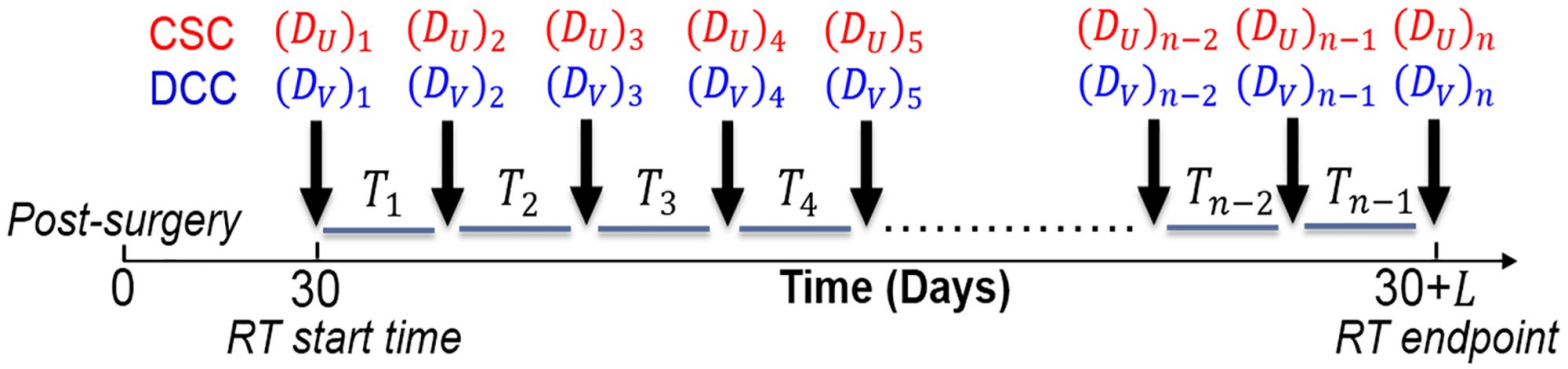
Schematic of optimization variables with respect to treatment time.

The optimization variables of interest are *D*_*U*_, *D*_*V*_, and *T*. *D*_*U*_ and *D*_*V*_ are vectors of length n, with each element (*D*_*U*_)_*i*_ and (*D*_*V*_)_*i*_ representing the dose applied to the CSC and DCC compartments during the *i*th dose fraction. *T* is a vector of length n-1, with each element *T*_*i*_ as the time interval between fractions *i* and *i* = 1. The total treatment duration is specified as *L*. A schematic of the optimization variables with respect to treatment time is shown in Fig 3.

The objective function is formulated in three terms. The first and main objective term, *W*(*t*_*eval*_|*D*_*U*_, *D*_*V*_, *T*, *X*), indicates the total tumor fraction U+V at the evaluation time point day *t*_*eval*_ given *D*_*U*_, *D*_*V*_, and T. Minimization of total cell number at a later time point delays disease recurrence. To reduce tumor burden during the treatment period, the second objective term that introduces time-weighted penalty on the total tumor fraction at each time point *t*, *W*(*t*|*D*_*U*_, *D*_*V*_, *T*), with *t* spanning from the beginning to end of specified treatment duration, was incorporated. The weighting of each evaluation time *t* is based on the corresponding remaining treatment duration to simulate the accumulation of tumor burden over time. The third term applies strong penalty when the total cell fraction *W* exceeds the defined disease recurrence total cell fraction *R*. *μ* and λ are weighting coefficients for the second and third objective terms.

Optimization constraints include total normal tissue biological effective dose (BED_normal_) to both compartments (*BED*_*U*_ and *BED*_*V*_), fractional dose limits (*D*_*min*_ and *D*_*max*_), time interval limits, and ratio constraint (*r*) between dose delivered to CSC and DCC to ensure plan deliverability. *L*_*S*_ indicates the lower bound of the time intervals, which was set to 1 to ensure at least one full day between all fractions. *α*/*β* represents the ratio between the linear and quadratic terms within the classic LQ model for surrounding normal brain tissue, which was set to 3 for all calculations.

The optimization problem was solved using a paired simulated annealing algorithm [38], in which a pair of elements within decided variable for change (T, *D*_*U*_, or *D*_*V*_) were changed in each iteration to maintain problem constraints. The problem was initialized at equal dose and time intervals for all fractions. For each iteration, a random number between 0 and 1 was generated as decision to vary either the time variable T or dose variables *D*_*U*_ and *D*_*V*_. The decision probability given to changing *T*, *D*_*U*_, *D*_*V*_ were 0.5, 0.25, and 0.25, respectively. A pair of elements in the decided variable were randomly selected to be varied, with both changes stepping in opposite directions to maintain equal total time or BED. To ensure that the optimized delivery times are feasible, the time intervals in T were maintained as integers by rounding the generated time step in each iteration. The change applied to the selected elements was sampled from a Gaussian distribution, with standard deviations specific to dose (*σ*_*D*_) or time (*σ*_*T*_) presented in Eq 5 below:

$$\sigma_{D}=\frac{s_{D}-1}{{\left(N_{D}+1\right)}^{1/T_{step}}}\;\sigma_{T}=1+\frac{s_{T}-1}{{\left(N_{T}+1\right)}^{1/T_{step}}},$$ (5)

where *S*_*D*_ and *S*_*T*_ are the step sizes at the beginning of the optimization, *N*_*D*_ and *N*_*T*_ are the number of times that a change in the dose and time were accepted. To account for the rounding procedure performed for time changes, *σ*_*T*_ was kept above 1 to ensure sufficient variation in T. *T*_*step*_ controls the decreasing rate of *σ*_*D*_ and *σ*_*T*_ as the number of acceptances increase. For dose changes, ratio constraints were applied by calculating the upper and lower bound specific to the opposite dose compartment corresponding to the same fractions. Cutoffs were applied to the generated changes if the resultant new value did not satisfy problem constraints. The objective function was evaluated after each iteration, and the change was accepted unconditionally if the objective function value decreased. If objective function value was not reduced, the change was accepted with conditional probabilities of *P*_*D*_ and *P*_*T*_ shown in Eq 6.

$$P_{D}=\frac{1}{{\left(N_{D}+1\right)}^{1/T_{prob}}}\;P_{T}=\frac{1}{{\left(N_{T}+1\right)}^{1/T_{prob}}},$$ (6)

where *N*_*D*_ and *N*_*T*_ were updated each time a dose or time change was accepted due to improvement in the objective function or the passing of conditional probabilities *P*_*D*_ and *P*_*T*_. 10000 total iterations were performed and the set of variables resulting in the best result was taken as the final optimization result. Outcome was assessed by the recurrence time point, the time at which the total cell number grows to 2.8×10^9^ cells, corresponding to radiographically noticeable volume increase of 1 ml in total tumor volume from the initialized postoperative volume. All optimization parameters are shown in Table 1. All calculations were performed in MATLAB 2013a (MathWorks, Natick, MA).

### II.4. Optimization scenarios

#### II.4.1. Optimization within conventional time frame

Optimization was performed within the equivalent duration, number of fractions, and BED_normal_ of a subset of currently utilized or previously applied GBM treatment fractionation schemes, including 2 Gy × 30, 1.8 Gy × 33, twice a day (b.i.d.) schedules of 1 Gy × 72 [6], 1.5 Gy × 40 [7], and hypo-fractionated approach of 5 Gy × 10 [14] to assess the potential in delaying recurrence with temporal dose optimization. For b.i.d fractionation schemes, the time increments were set in units of half days and *D*_*min*_ was lowered to 0.5 Gy. The objective function evaluation time point (*t*_*eval*_) was set to 300 days. The recurrence time resulting from optimization was compared with the recurrence time of the original dose fractionations predicted by the model.

#### II.4.2. Super hyperfractionated regular schedules

Varying dose fractionation within the conventional treatment time frames has been shown to modestly impact the outcome of GBM radiotherapy. The potential in further delaying recurrence time with a super hyperfractionated treatment approach was therefore tested. Specifically, the potential in improving outcome with the novel approach of treating GBM with a protracted schedule was explored with a total treatment course of up to one year. Simulated annealing optimization on dose was performed with fixed time points of weekly, bi-weekly, and monthly, with total BED_normal_ of 100 Gy (equivalent to that of 2 Gy × 30, assuming α/β = 3). The objective function evaluation time point (*t*_*eval*_) was set to 500 days. The optimized recurrence results were compared with that of equal dose throughout all fractions for all time schedules.

#### II.4.3. Super hyperfractionated temporal dose optimization

Full optimization with both time and dose as variables was performed with number of fractions equivalent to weekly, biweekly, and monthly over one year. The objective function evaluation time point (*t*_*eval*_) was set to 500 days. The resultant recurrence times were also compared with corresponding regular time schedules with equal dose over time. To assess the synergy of dose escalation and hyperfractionation, the outcome with dose escalation to BED_normal_ of 150 Gy (equivalent to 2 Gy × 45, assuming α/β = 3) was also generated.

#### II.4.4. Robustness of the model to varying input CSC radiobiological parameters

To understand the robustness of simulation to input parameters, particularly the radiobiological parameters of CSC that may show a large uncertainty, we performed a sensitivity study for varying *α*_*CSC*_ and β_*CSC*_ values. The ranges of *α*_*CSC*_ and β_*CSC*_ were set to be 0.01 to 0.02, and 1e-07 1e-04, respectively.

## III. Results

### III.1. Optimization within conventional time frame

The resultant recurrence time from optimizing within conventional time frames is shown in Table 2. The recurrence time point of the conventional 2 Gy × 30 delivery predicted by the model is 250.3 days, in close agreement with the observed average recurrence time of 7–9 months [35, 39]. Variation in time and dose did not significantly improve the recurrence time for all attempted historically and currently administered fractionation schemes. The improvement in overall recurrence time is slightly greater for 1 Gy × 72, which has the longest treatment duration, indicating that an extension in the treatment duration might help improve the result.

**Table 2:** Conventional fractionation optimization results.

Equivalent Fractionation scheme	Total duration L (days)	BED_U,V_(Gy)	Original recurrence (days)	Optimized recurrence (days)
**2 Gy × 30**	39	100	250.3	254.7
**1.8 Gy × 33**	44	95.04	247.6	251.3
**1 Gy × 72 B.I.D.**	49.5	96	258.2	269.1
**1.5 Gy × 40 B.I.D.**	25.5	90	249.4	255.5
**5 Gy × 10**	11	133.33	234.4	234.5

### III.2. Super hyperfractionated year-long regular and variable schedules

**Fig 4:**
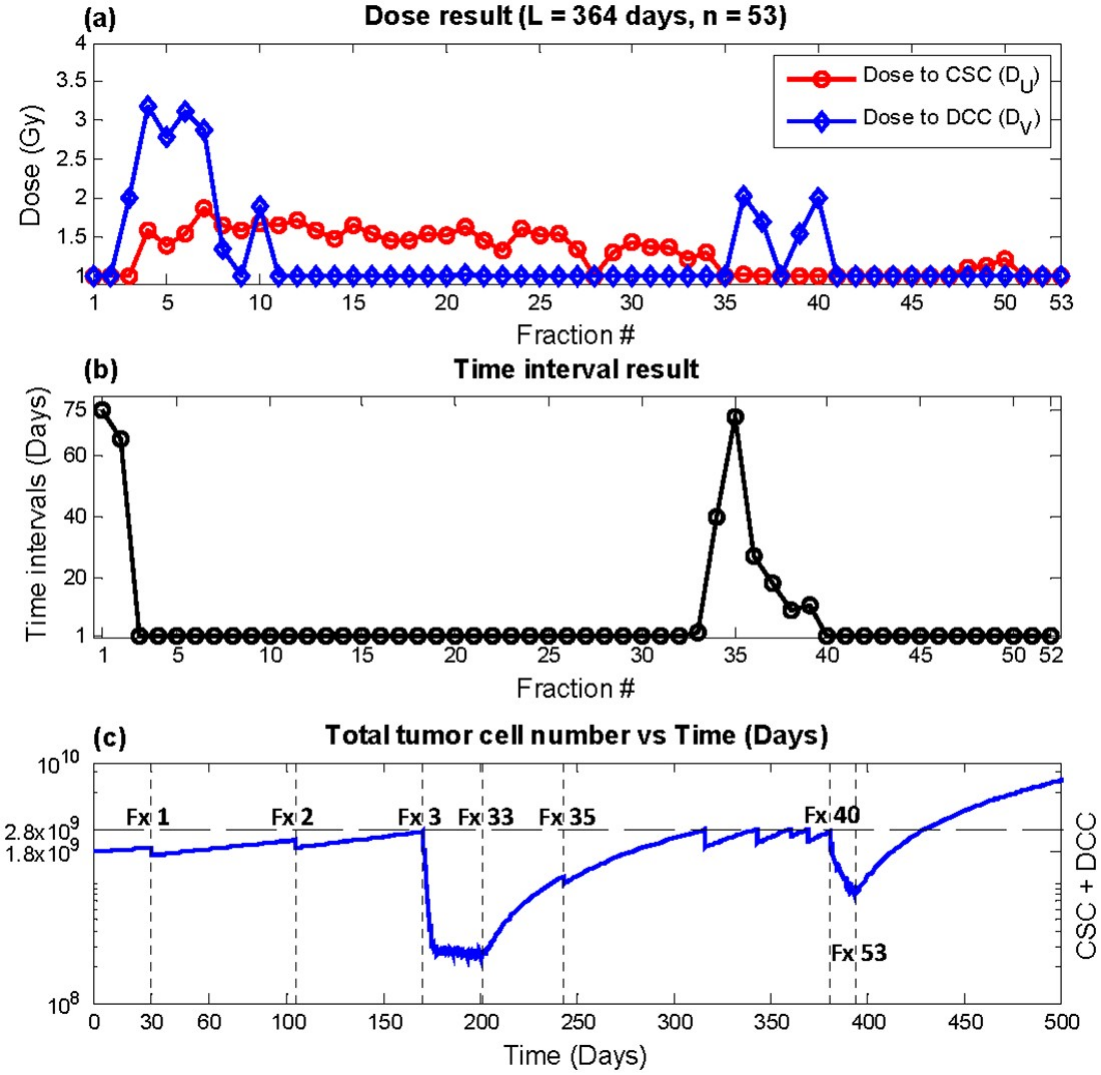
Optimization result, total duration *L* = 364 days, number of fractions *n* = 53 (weekly equivalent). (a) *D*_*U*_ (red circles) and *D*_*V*_ (blue diamonds) (b) Time interval *T*(c) Total tumor cells vs. time. Recurrence time with this plan was predicted to be 430.5 days.

**Fig 5:**
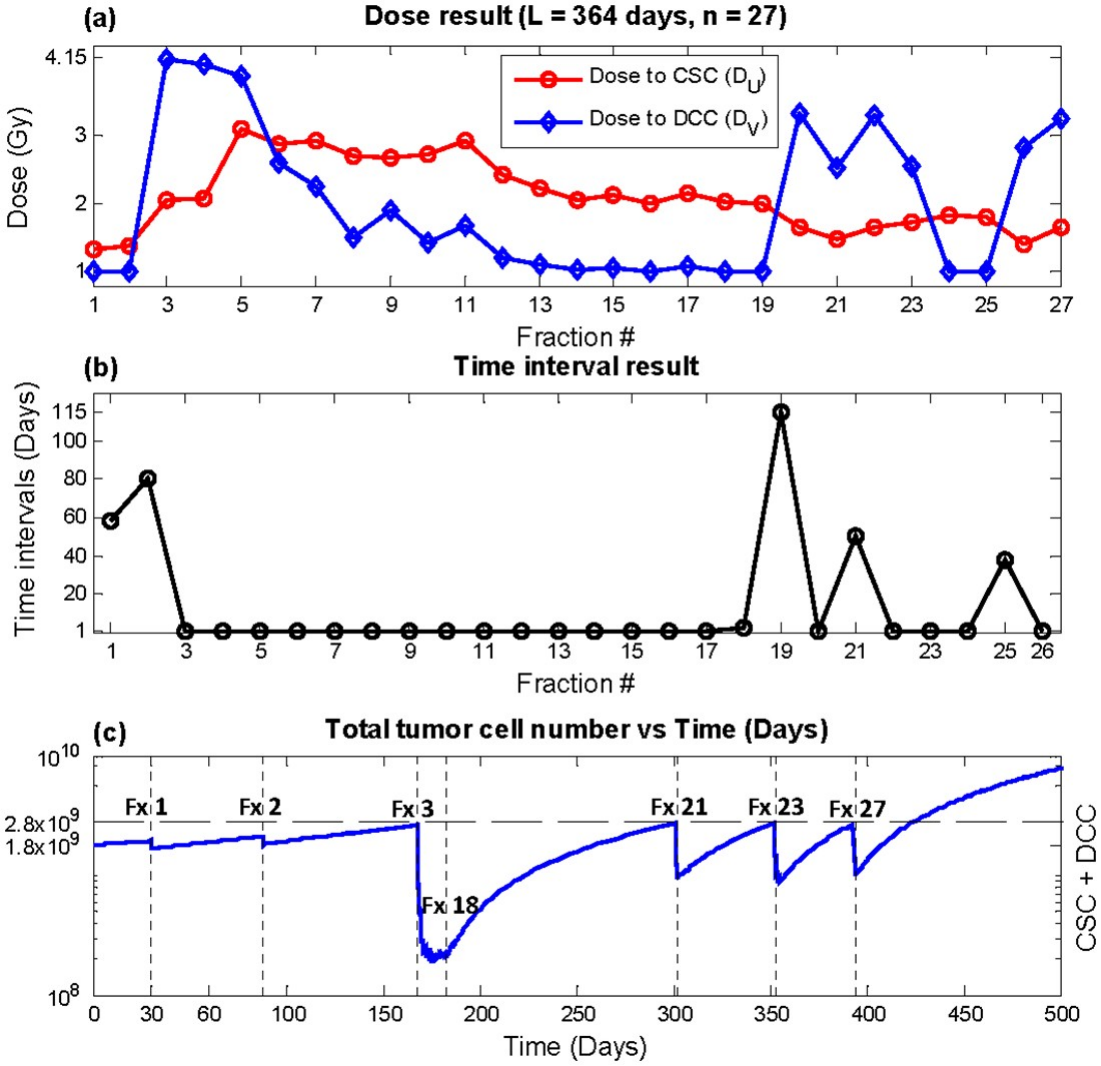
Optimization result, total duration *L* = 364 days, number of fractions *n* = 27 (bi-weekly equivalent). (a) *D*_*U*_ (red circles) and *D*_*V*_ (blue diamonds) (b) time interval *T* (c) Total tumor cells vs. time. Recurrence time with this plan was predicted to be 423.9 days.

**Fig 6:**
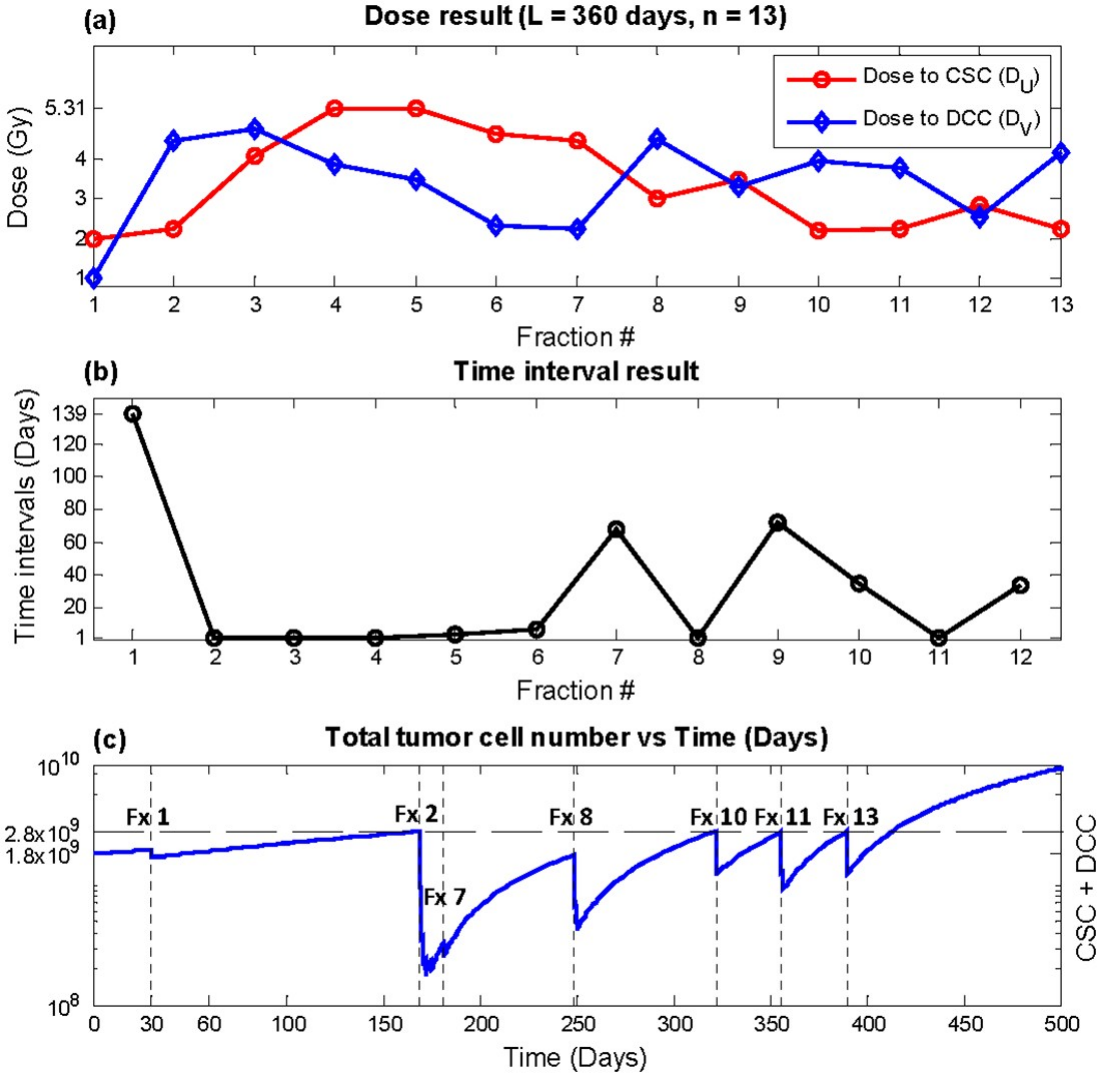
Optimization result, total duration *L* = 360 days, number of fractions *n* = 13 (monthly equivalent). (a) *D*_*U*_ (red circles) and *D*_*V*_ (blue diamonds) (b) time interval *T* (c) Total tumor cells vs. time. Recurrence time with this plan was predicted to be 413.3 days.

Outcome from optimizing super hyperfractionated schedules equivalent to year-long weekly, bi-weekly, and monthly treatment with fixed and variable times are shown in Table 3. Within Table 3, the column labeled “Constant” indicates equal dose and time interval throughout the year, “fixed time” results from holding constant time intervals while optimizing doses, and “variable time” shows outcome with doses and time intervals all as optimization variables. With BED_normal_ equivalent to that of the conventional 2 Gy × 30 treatment, which had recurrence time of 250.3 days, all three super hyperfractionated year-long regular schedules with equal dose (“Constant”) significantly delayed recurrence by more than 70 days. Optimization of time intervals in conjunction with doses further postponed recurrence by more than 2 months from the regular fixed dose schedules. The weekly equivalent plan with 53 fractions, as shown in Fig 4, achieved the largest benefit of 180 days compared with conventional therapy using 2Gy × 30. The time interval result (Fig 4b) indicates relatively infrequent treatments in the beginning, followed by aggressive once per day delivery in the middle of the treatment course, where the tumor size is significantly reduced. With an appreciably smaller tumor, the treatment again becomes infrequent, until the tumor size approaches the recurrence level, where an increase in treatment frequency is observed. The rate of treatment continues to increase up to the end of the treatment course in order to complete the treatment with the lowest possible tumor size while keeping total tumor size under the defined recurrence level. In terms of dose, *D*_*U*_ was relatively constant throughout time, while *D*_*V*_ was held at minimum dose of 1 Gy for most fractions and peaking at the fractions immediately following larger time intervals (Fig 4a). The dose and time results of the bi-weekly (n = 27) and monthly (n = 13) equivalent plans are shown in Figs 5 and 6, respectively. The trend in outcome of less aggressive fractionation in the beginning of treatment, preceded by dense fractions in the middle of the treatment course, followed by a decrease in frequency, and then a final increase in treatment aggressiveness was also observed. Dose optimization alone achieved substantial benefit as well but less than variable time as expected, as demonstrated in the column labeled “fixed time” in Table 3.

**Table 3:** Super hyperfractionated year-long optimization results.

	Equivalent Fractionation scheme	Total duration *L* (days)	*BED*_*U*,*V*_(Gy)	Recurrence time (days)		
				Constant	Fixed time	Variable time
**Weekly**	1.3125 Gy × 53	364	100	372.4	401.2	**430.5**
**Bi-weekly**	2.1553 Gy × 27	364	100	351.1	403.7	**423.9**
**Monthly**	3.5325 Gy × 13	360	100	322.2	411.8	**413.3**

**Fig 7:**
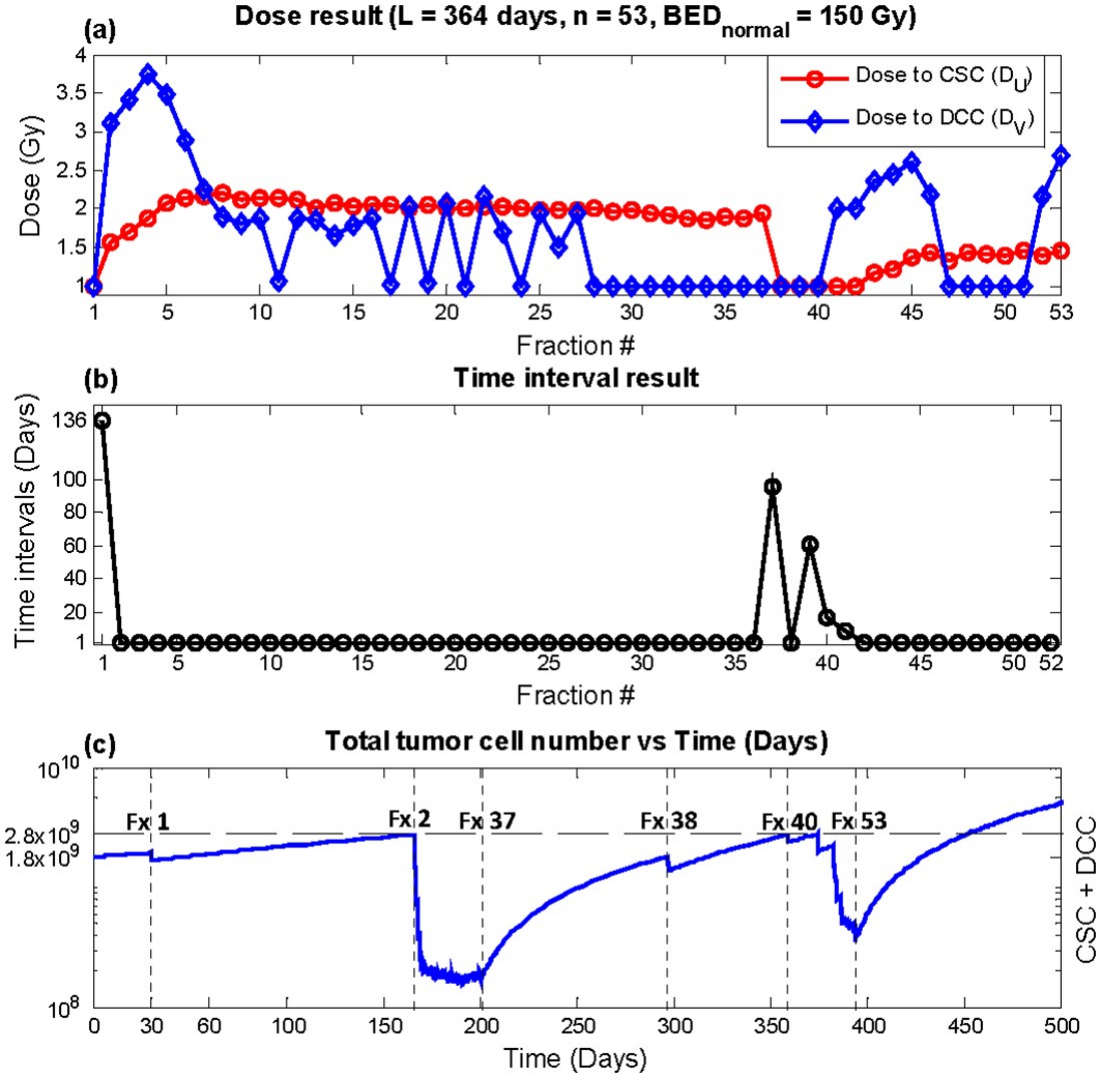
Dose escalation (BED_normal_ = 150 Gy) optimization result, total duration *L* = 364 days, number of fractions *n* = 53 (weekly equivalent). (a) *D*_*U*_ (red circles) and *D*_*V*_ (blue diamonds) (b) time interval *T* (c) Total tumor cells vs. time. Recurrence time with this plan was predicted to be 452 days.

The result from escalating BED_normal_ to 150 Gy is shown in Table 4. Maximum recurrence time was also observed for the weekly equivalent plan at 452 days, which provides a 201 days delay in recurrence compared with conventional delivery. The resultant plan from the weekly equivalent optimization with dose escalation is shown in Fig 7. In terms of time intervals, trends similar to the results without dose escalation was observed. However, oscillations between one and two Gy was shown for *D*_*V*_, unlike the stable one Gy dose fractions shown in Fig 4a. For the bi-weekly and monthly optimizations, dose escalation did not alter the general trend of the result. It is important to note that although the tumor cell counts approach the threshold for recurrence, it is the result of optimization to deliver treatment fractions when the impact is maximized. The comparison between different treatment regimen should be made only after the entire treatment of radiation dose, which is dictated by normal tissue tolerance, is delivered. Instead, the model robustness is shown below as the time recurrence vs. varying radiosensitivity parameters.

**Table 4:** Super hyperfractionated year-long optimization with dose escalation.

	Equivalent Fractionation scheme	Total duration *L* (days)	*BED*_*U*,*V*_(Gy)	Recurrence time (days)		
				Constant	Fixed time	Variable time
**Weekly**	1.7773 Gy × 53	364	150	407.6	413.6	**452.0**
**Bi-weekly**	2.8493 Gy × 27	364	150	406.1	412.7	**441.3**
**Monthly**	4.5716 Gy × 13	360	150	325.7	410.2	**424.5**

### III.3. Model robustness

Table 5 shows the predicted recurrence time for varying input CSC radiobiological parameters. The recurrent time is stable for up to 40% and 1000X variation in *α*_*CSC*_ and *β*_*CSC*_, respectively.

**Table 5:** Predicted recurrent times as a function of the input CSC radiobiological parameters.

	**Equivalent Fractionation**	**Recurrence Time (days)**					
		**αCSC = 0.01**	**0.012**	**0.014**	**0.016**	**0.018**	**0.02**
**Weekly**	1.3125 Gy × 53	432.05	432.00	431.25	446.98	449.01	462.14
**Bi-weekly**	2.1553 Gy × 27	414.02	426.23	429.07	447.36	445.03	452.33
**Monthly**	3.5325 Gy × 13	415.03	422.48	420.24	431.39	425.88	428.58

	**Equivalent Fractionation**	**Recurrence time (days)**							
		**βCSC = 1e-07**	**1.77e-07**	**2e-07**	**3e-07**	**5e-07**	**1e-06**	**1e-05**	**1e-04**
**Weekly**	1.3125 Gy × 53	430.62	427.51	432.76	432.16	428.84	428.80	433.43	429.81
**Bi-weekly**	2.1553 Gy × 27	431.73	429.33	426.44	431.53	431.23	419.21	430.95	419.87
**Monthly**	3.5325 Gy × 13	413.88	415.50	416.65	417.38	415.44	414.89	418.92	415.80

## IV. Discussion

Without assuming different radiobiology than the well tested linear-quadratic model, the previously proposed dual compartment ODE model [28] was the first radiobiological model to our knowledge capable of reconciling the perpetual radioresistance in patient and the apparent moderate radiosensitivity *in vitro* of human GBM, therefore providing us with a theoretical platform in exploring the potential in delaying GBM recurrence with differing dose fractionation schemes. The radiobiological model is separate from other potential microenvironment related drivers, e.g., hypoxia. Therefore, our model could complement existing GBM research focusing on different mechanisms. We extend the model into an optimization formulation allowing for optimization of dosing temporal fractions. Previously work focused on optimizing within the confinement of conventional once or twice per weekday treatment times, and significant improvements in outcome were shown [21–23, 40]. However, the unique pattern of aggressive recurrence of GBM leads to considerably different dose fractionation strategies. As shown in our study, optimization within the conventional time frame was ineffective in substantially delaying disease recurrence, which therefore inspired the idea of treating GBM with a prolonged super hyperfractionated approach. The protracted treatment duration, along with dose fractionation optimization, resulted in recurrence delay of up to 180 days. With dose escalation to BED_normal_ of 150 Gy, which is substantially lower than the BED_normal_ of combined external beam and brachytherapy therapy trial previously conducted [18, 19], the recurrence time point was further delayed to 452 days from the simulated postoperative time point. Although still not a cure, the predicted delay is not trivial in reference to one of the most effective chemotherapy for GBM by temozolomide [1] that was shown to improve median survival by 3 months. Besides the potential survival benefit, our robustness study showed that the potential survival benefit from the proposed optimized hyperfractionation is robust to variations in the stem cell radiosensitivity parameters.

Another interesting observation is that our results suggest super hyperfractionated treatment to be carried out in four cycles with varying strategies. The first treatment cycle consists of low and infrequent dose fractions, just enough to maintain the total tumor cell number below the recurrence level. The second cycle applies aggressive once per day treatments beginning with larger dose fractions that gradually decreases and stabilizes at a lower level. The compacted therapy quickly reduces the total number of tumor cells before moving into the third stage, without depleting the total allocated BED_normal_. The third cycle again uses fractions spaced farther apart, mainly to maintain the total number of cells. The final phase is characterized by another series of densely spaced treatment fractions to minimize the total cell numbers as much as possible before the end of radiation therapy.

The simulated annealing (SA) optimization algorithm generally applies only one variable change within each iteration [38]. The one variable approach required a constraint check after each iteration and resulted in early local minimum convergence due to the difficulty in finding additional answers that satisfy problem constraints. The novel pair-wise opposite step size approach we have presented for this problem helped maintain the random-walk search within the time and dose domain that satisfies problem constraints, contributing to increased optimization efficiency and results far superior to that of one variable SA approach. This method can also be utilized on many other applications when the optimization constraints are not straightforward.

There are several limitations with the study. Although the model was able to successfully reproduce the aggressive regrowth of GBM after aggressive treatment, it does not take into consideration biological factors such as tumor vasculature, oxygen content, the effect of asymmetric divisions, and spatial heterogeneity. Modeling tumor microenvironment may render the model more realistic but also increase the complexity of modeling and the uncertainty from estimating additional model parameters. Modeling the intratumoral heterogeneity may improve our capability of predicting treatment response and optimize the treatment fractionation but this study still highly simplifies an actual tumor. Rigorously designed preclinical and clinical studies are needed to test the mathematical model prediction. The validity of the simulation can depend on the accuracy of input parameters, which were estimated based on average recurrent time and the volume of recurrent tumors. Based on our sensitivity study, the simulation results are stable to large CSC radiobiological input parameter changes. Furthermore, to further minimize the residual uncertainties, in the future workflow, it is possible to perform in vitro assays on the surgical specimen, including DNA expression, flow cytometry, and radiobiological survival on the cancer cells. The treatment regimen can then be personalized according to the individual assays.

## V. Conclusion

A temporal dose fractionation optimization in the context of cancer stem cell dynamics and heterogeneous radiosensitivity within GBM was introduced. The model demonstrated that substantial delay in GBM recurrence could be attained with a super hyperfractionated treatment approach. Further testing is needed to validate the efficacy of this novel treatment method.
